# Report of Trial for Mal-Practice

**Published:** 1848-08

**Authors:** 


// BUFFALO MEDICAL JOURNAL

AND

MONTHLY REVIEW.

VOL. 4. AUGUST, 1848. NO. 3.

### ORIGINAL COMMUNICATIONS.

ART. I.—*Report of Trial for Mal-practice.* WILLIAM TIMS, *vs.* JAMES P. WHITE. Supreme Court: Erie County Circuit, June 26th, 1848—Mr. Justice JAMES G. HOYT, presiding. *E. Cook*, for plaintiff; *J. G. Masten*, *S. G. Haven*, and *James Sheldon*, for defence.

[The following condensed report is made from minutes of defendant's counsel.]

Mr. COOK—to the Jury. This is a special action on the case, brought by the plaintiff against Dr. White. The gist of the action is, that defendant, by treatment of right thigh bone, which was broken in the fall of 1843, acted so negligently and unskillfully, that, by means thereof, the thigh became deformed. The cause has been twice tried;—once in 1844, and in June 1845, and the Juries did not agree upon a verdict.

The facts are these: In December, 1843, plaintiff was engaged on a building, and fell and fractured the bone of the thigh at about the middle section, not exactly transeverse, perhaps, but slightly oblique. A surgeon was sent for. Dr. White examined the limb, and declared it to be an ordinary, simple, transverse fracture. He put it in a fracture-box, then journeyed East, and was absent for some days. We say it was not attended to as it should have been, and we claim it was not properly reduced; i. e. the bones were not properly put in apposition.

Plaintiff will show that when the limb came out of the fracture-box, it was not straight; and you will see that his limb is curved, and there is also a protuberance. We ask you to find that this happened through the carelessness of defendant.

A fracture is a simple thing to cure. It will heal itself; but during the healing process, great care and attention is required to prevent the parts from *lapping* while the limb is in the box. There are no arbitrary rules upon the subject. It must be left to the discretion and good judgement of the Surgeon to treat this as he may think proper. We shall show you that the limb was not properly dressed; the proper fracture-box was not used; the proper splints were not put on; and we claim a verdict for the wrong acts of the defendent, if you shall find it to be his fault.

SAMUEL HILLIARD was then sworn for the plaintiff:—Knows the parties to the suit, married the sister of the plaintiff. The plaintiff lived in the fall and winter of 1843 on Main st. about a mile from the Eastern R. R. Depot. At the shingling of this depot, on the 2d day of December, 1843, the plaintiff slipped from the roof and had his thigh broken. The break was across there, (witness describes about middle of thigh bone); I told him it was a good break, as I discovered it was very near across and a straight break. I accompanied the men who carried him, as far as Main and Swan sts., and then I went on. Before starting we inquired who was the best surgeon. Some said Dr. White, and some said others. Near Swan st. I met plaintiff's father, who started from depot ahead. He had been after Dr. White, and said he was not at home, and was then going after Dr. Sprague. To prepare the way, I went on to get his wife, who was a sickly woman, away, and to get my wife there.

About the time plaintiff arrived, Drs. White and Sprague both arrived; I think Dr. S. was first. Dr. W. took charge of the patient, made examination of leg, and said, I believe the terms were,—it is a simple fracture. Plaintiff was now taken into the house and laid into bed, and they prepared to dress the limb. Dr. W. turned to Dr. S. and said, I think, he should use Sir Astley Cooper's inclined plane, asking whether it was the best one or not, or whether he used that one. Dr. S. answered, in some cases he used one kind, and in some another. Some conversation ensued as to who should have the patient, and I believe Dr. W. claimed the precedence, as he was first called. I returned to my work, and on my return at evening, the plaintiff was lying with his leg in a fracture-box, wanting the foot-board. The knee was slightly raised; he was laying upon his back. I made a shield of hoops to uphold the bed-clothes from the limb, and remained there all night, I think; was generally there after this during the evenings, and several times during the day, or in the morning, as I lived near by; and I believe I sat up with him the first, second and third nights after the injury. After that, his brother James Tims and Mr. George Parker sat up with him nights. There may have been an evening I was not there, up to the time the limb came out of the fracture-box. Not to my actual knowledge was the position of the leg in the box changed in all this time, save once when Dr. White took it out. I had seen the limb the night before in the box, at Noon, I saw it out. In the evening, probably eight o'clock, I gave the limb an examination. There was a very great curve in the thigh bone, the flesh appeared shrivelled, and a large lump on the outside. The clothes were off and I rubbed it with my right hand, for he complained of pain in his knee and limb. I told my wife, when I went home, the limb was crooked. I saw him again that evening. Mr. Parker, Mr. Tims' father, and brother James, and I think Mr. Haddock, were present, but am not certain. They all examined it, but I don't think the subject of the crookedness of the limb was mentioned aloud while I was there. It was about a Sunday, a fortnight, before I saw it again. I had been sick and confined to my bed, and he came to my house on crutches. It then presented the same appearance. I next saw it on Tuesday following at his house. Dr. White, Mrs. Tims and Sarah Maloy we.e present. Dr. White said it was a bad job; that it had been curved by muscular contraction or something of that kind. At this time Dr. W. fitted a splint on the outside and bandaged it, saying it would have a tendency to strengthen it and prevent its going any farther. I remarked the limb was crooked, when it came out of the box. He said that was a mistake, I was mistaken, or something to that effect.

I was not present when it was taken out of the box—I saw it in the box one day and the next it was out. I think it was Wednesday that it was in and on Thursday it was out. On Friday, or day after, I made effort to get the patient up; I moved him round to the side of the bed, when he complained the pain was so great that I let him rest; one leg was over the bed, but not so as to descend any, the broken leg was on the outside. The limb was naked, dark colored, skin not broken.

John Tims, father of plaintiff, sworn:——testified substantially as did Hilliard, and examined the leg on the second night (Friday) after it came from the box, for the first time and saw "a pretty good difference" between it and the other. I looked at it and satisfied myself it was crooked—no one said to me it was crooked before Friday night—his complaint of pain was general but never so severe as to my knowledge require sending for the doctor. I asked him how he was on first Friday night after the box was taken off, and learned he was middling well. His well leg is a straight leg, but a little hollow. Three or four days after Friday the pain came on and was very great, and continued two, three or four days. I never told

Alanson Webster that the leg came out of the box straight. I know Gilbert Hoyt. I never told Hoyt after leg was taken out that William had been using it too much, and that he had a pull back. The box did not come up so far as to protect the whole length of the thigh bone underneath—there were no splints on the top of the leg, inside splints merely.

George Parker, sworn:—My sister is wife of plaintiff—resided in upper part of same house—did not see the limb set, but saw him same evening; sat up with him part of one or two first nights after—was present and examined limb first night after limb was removed from the fracture-box—rubbed it with liniment. I looked at it to see if it was crooked, but cannot say whether it was that night or next night. Old Mr. Tims remarked that he thought it was crooked this second night, I looked at it then and I thought it was. I have never heard him complain a great deal of pain until after leg was taken out of box. The outside splint, while leg was in the box, did not set close at the upper end, but projected so that I could get my fingers beneath—[otherwise, the dressing was such as other witnesses described.]

Elizabeth Hilliard, sister of plaintiff—was present at the dressing about ten days after the injury. Doctor Wilcox and Charles Deer assisted Dr. White. Was not present at the final removal from the box, but in course of same day, (Thursday) went over and saw it was out. It had a shrivelled appearance and was swelled on the out side—I did not notice whether it was crooked. The next day I rubbed it, it did not look straight; my husband had before told me it was crooked. On Saturday Dr. White was present and my brother was in great pain—the doctor did not prescribe anything, or look at the limb, but said it would be off in a day or so. Nothing was said about my brother's trying to get up, to Dr. White.

Joseph G. Haddock sworn: saw him occasionally when confined with broken limb—saw him the next Sabbath after the limb came out of the box, about the 15 or 17th January, and noticed the limb was crooked; he was on the bed; he had his pantaloons on I believe; did not feel the thigh.

James Tims, brother of plaintiff, testified to the points at issue, much as witness first called.

Sarah Tims, (formerly Sarah Maloy) sworn:—Wife of John Tims, Jr. Lived in family of William when his leg was broken and under treatment; was in the house, and soon in the room, after the box was finally removed. On next day (Friday) was sent for Dr. White, on account of plaintiff's suffering pain—did not see Dr. W.—but his student, Dr. Newman—Dr. White did not call that day. The first I remember of seeing Dr. W. there, after the limb was out, was on Sunday, eight or ten days after—he came in walking up to Tims, laid his hand on his thigh and said: why Tims you have got a crooked leg—that was all I heard. On first Saturday evening, six o'clock, after the limb was taken from box, I came home and found plaintiff sitting up by the bed in a chair; don't know who helped him up; Mrs. Tims was sickly and unable to lift him up, and the children were not large enough; I don't know how long he set up; I stayed long enough to have my supper in the room; can't say I saw him up on Sunday; I think Mr. Haddock came in on Sunday, and said, he must not set up.

Dr. Josiah Trowbridge sworn: Is a practicing physician; was formerly a practicing surgeon, for about 25 years; I relinquished that practice because the legislature have enacted, that if any person is convicted of being engaged in acquiring a knowledge of Anatomy and Surgery by dissection, (and it is the only way in which a perfect knowledge can be acquired) he shall be sent to the State Prison. Our Courts and Juries have decided that if any Surgeon does not possess this information, (or otherwise, if he commits an error) he shall be mulcted with ruinous damages. I resolved therefore not to serve the public on such conditions.*

In the treatment of a transverse fracture of the middle section of a thigh-bone, I should think it sufficient to put it upon a double inclned plane, after putting the bones in apposition, and applying simple short splints, these to be secured by bandage or tapes; if much inflammation or swelling, it is common to apply whiskey or water. Having called upon the patient once or twice previously, to see if comfortable and bones are in place, on the 8th or 10th—6th to 10th day, if a man age 28 and of good habits, from the time of reduction of fracture, I should make the second dressing. The difference between a transverse and oblique fracture could be ascertained by a surgeon before, or certainly at time of making reduction. If at time of making second dressing, I found all in good condition, I should not make any change, but continue the splints, &c. The object of the whole is to keep the bones in apposition. There is not ordinarily

* In answer to the question direct of counsel, these remarks elicited from the oldest surgeon called to the stand, and one who is noted for the firmness with which he has ever upheld the true dignity of his profession, have their significance, and lose naught of their pertinency when to them we add,—in this city there are but few surgeons of years, or of reputation in the profession, who have not been latterly crowned with the accompanying honors of a public prosecution for mal-practice! Dr. T., by his timely and judicious course in laying down the scalpel, although a shining mark, we believe thus far has escaped the attempts of these modern, prolific and hydra-headed *Chirurgophagi*. If such trials result elsewhere as they have here, how fast soever they may increase in numbers, these feeders-on-surgeons must soon become attenuated to mere shadows by want of success in fastening upon their prey. W T.

any union of the bones up to the 8th or 10th day. About that time the callus begins to form, and now our object is to see that all is placed right for a permanent dressing. I have seen several fractures of the thigh, and treated them. When the leg is put into the fracture box, the box should come far enough up to protect the knee, keep the knee at the angle; you cannot get the box higher than the ischdela-lum, the object is to make extension at the knee, the splints guard against displacement and the box makes the extension. I used to put short splints on laterally, and as a matter of precaution, sometimes one on the top of the thigh. The average time for keeping the limb in the fracture box, is about six weeks, where there has been no untoward circumstances, though in a patient like Mr. Tims I used to keep it on about seven weeks, but that is not the average; if a splint should protude so that I could get my hand under it, I should look to the cause and rectify it. Each case must depend upon its peculiar circumstances in determining about the time of removing the dressings. I should remove the bandages and short splints and examine with gentle flexion, to see if bony union had taken place, in order to decide upon removing the box. Here the judgment of the Surgeon is very important. I would not remove the box until I thought the patient might moderately move his leg in bed. My impression is, I would keep the short splints on a short time after the box was removed as a matter of precaution—though not important. It is proper to remove the splints as soon as possible, to let the blood circulate, &c. (Witness examines plaintiff's leg.) I think the bones are not perfectly in apposition and that causes the curve. I should think the bones might assume their present condition after their coming out of box, in apposition, and after a sufficient ossification to authorize me to remove the limb from box, in consequence of some violence. Spasm of the muscles,—any violence, sudden hanging of the leg over the bed, or chair, which was too high to rest the foot upon the floor would do it. If the bones come out of box in their present condition, I should think they either lapped, or were thrown out of line. I should hardly think they were out of line enough to produce a shortening of one inch. When bony union is sufficient to justify removal of box, the bones cannot slide by, or lap, it would be the same as breaking the leg and cause about as much pain as the original fracture, though if broken a week after, I should not apprehend any particular danger. The bony union is not strong enough to bear the weight of a man without crutches, we therefore put them on crutches.

I cannot say whether plaintiff's leg is a transverse or oblique fracture, from my examination I should say it is more than half an inch shorter.

I did not find it so short as I expected; his other leg is more curved than thighs in men generally are. I don't think Tims' leg is a good leg; but it is a medium cure. I should expect a better leg if the Surgeon and patient both did their duty, and all was favorable.

[A series of questions written by defendant were put: to these are subjoined the answers of this witness.]

Does a sudden snap or grating noise, in transverse or slightly oblique fractures of the thigh bone, accompanied by disappearance of the deformity, afford strong evidence that the fracture is reduced? *Ans.*—It does. I should have no doubt about it in such a case.

If a limb had been well dressed and fitted in this box, would the ends of the bones have been likely to become displaced without the foot-board being applied during the cucceeding six hours? *Ans.*—I should think not.

Would there have been any danger of displacement, in carefully applying the foot-board at that time? *Ans.*—I should think not.

If the bones had been displaced, would it have aggravated the sufferings of the patient during the succeeding ten or twelve days? *Ans.*—I should think it would for some few days.

If any displacement has taken place, can it be rectified at the dressing on the tenth or twelfth days without detriment to the fracture? *Ans.*—It can.

If in a simple fracture, a healthy man, 28 years of age, you had carefully adjusted the ends of the bones, and the bandages, on the tenth or twelfth day, and no untoward symptoms should supervene, at what time would you expect to be able to remove the apparatus? *Ans.*—In six weeks, provided I had confidence in the patient.

If under these circumstances you were to remove all the splints on the 47th day of the fracture, and everything appeared favorable, would you consider it safe and prudent to leave them off, directing the patient to remain in bed two or three weeks, and gently flex and extend the limb? *Ans.*—I should provided the patient followed my directions.

If under the above mentioned circumstances, you were to call on the following day, or 48th after the fracture, and find on careful examination, everything favorable, would you consider it judicious and prudent to dismiss the patient with proper directions for taking care of himself? *Ans.*—I should.

Suppose on the same evening he were to send for you, and on your calling the following morning he should say, "Doctor, I had considerable pain last night but am easier this morning, and I have sent for you to know if it is common for such pains to come on after the box is off." Would you think there was anything unusual, likely to be present? *Ans.*—That would depend entirely upon circumstances. If a slight pain, I should think there was nothing out of the way; but if very severe, it would be likely to attract my attention.

Would you think it necessary under these circumstances to do more than prescribe quietude, gentle flexion of the knee in bed, and friction with a liniment? *Ans.*—The answer would be the same as before.

Do you believe the callus flexible at a time when it is safe to dismiss the patient? *Ans.*—I should think it was if sufficient force was applied.

Do you believe that spasmodic action of the muscles *could* bend the callous from the 50 to the 60th day? *Ans.*—I believe they could do it.

Would too early use of the limb, after removal of bandages, be likely to excite irregular or spasmodic action of the muscles? *Ans.*—I cannot say that it would have that effect. I have never seen any effect of the sort, that I know of.

If called to a patient with a curvature in a callus of the thigh bone, at how late a date would you think it prudent to undertake to straighten it by means of apparatus? *Ans.*—It would depend upon circumstances. In a a patient of the age of 28, it might be, I should think, safe to attempt it as late as the tenth or twelfth week.

If the present deformity in Tims' leg had existed at the time it was in splints, what would have been the result of the firm application of this splint over the prominence for six weeks? *Ans.*—Either one or both ends would have been raised from the thigh, and it would have produced irritation at least; and, if proper force had been applied, ulceration probably.

What is your opinion of the present condition of Tims' leg? *Ans.*—It is not near as bad a leg as I have seen, from the same kind of fracture, nor as good. It is about a medium cure of leg.

Does more or less deformity usually follow fracture of the thigh bone? *Ans.*—It does even in the best cases.

Does the shortening of the limb one inch neccessarily imply unskillful Surgery? *Ans.*—It does not necessarily, for it may arise from causes beyond his control.

I have never read Chelius on Surgery; the author I have heard well spoken of, but the publisher and commentator, South, I have not heard so well spoken of.

Dr. Charles Winne, sworn: I should make my dressing at time of reduction a permanent one, and afterwards only give care and attention; using four splints and a roller and many tailed bandage if fracture transverse, without extension or counter extension; if oblique I would add the double crutch splint or apparatus of Dr. Gibson. My own experience with it has satisfied me that it is better than some other apparatus that I have used.

After limb thus dressed, I should deem it necessary to visit in the course of a few hours, to learn of comfort of patient, and stability of bandages, &c. For first two or three days I should call twice a day; if doing well I should call twice a day—as the bed may sink, the bandages loosen, and other matters of about as much importance as the setting of the bone would require attention. While the callus was forming I would call once a day, and keep so calling, until the callus was judged so formed as not to need such frequent calls; after this perhaps twice a week. I should make examination by removal of dressings as early as first 24 hours, if all was not right, and perhaps continue to examine it till I was satisfied the callus was formed.

As to final removal of splints, I should be guided by authority, &c., say six weeks bound pretty firmly, and afterwards more loosely, or apply some light ones, as felt or pasteboard, to protect the limb in its weak state. I should judge by moving the limb, and the pain, if any, &c., and an exercise of my own judgment, whether the callus was sufficiently formed to remove apparatus and splints.

In the latter calls, I would examine the limb of the patient; I would untie the bandages and take off the splints generally, and determine if every thing was right. It takes but very little time to do it.

[In general, the opinion of Dr. Trowbridge, as to present condition of bone, and cases supposed hypothetically, or otherwise, were coincided in.]

Of Chelius on Surgery, I can only speak from its reputation—the best from any German author. By eminent reviewers it is said to be elaborate, and the commentaries of South, as being very able; his reputation is that of an able surgeon, and his text of as high authority as Chelius himself. If they clash, would prefer South; am not particularly acquainted with either author.

The box present, I think is Sir A. Cooper's. It is in very general use among surgeons. I think Gibson's apparatus better, in some cases, certainly; know of no surgeon in town who practices my method. Where the apparatus was taken off after 48 days, the patient ought not to get up, even if callus is hard, by his own exertion. It would be very bad on the part of the patient. I have treated three fractured thigh bones myself, and seen several.

I know Dr. Wilcox. He is a good physician and surgeon; don't know as I knew him in 1843, and how good he might be then. I know Mr. Lane, who read with Drs. Loomis and Sprague; he was a diligent student and a worthy young man.

Dr. B. BURWELL testifies:—I am a practicing physician in this city; practice surgery, not enough now to be called a prarticing surgeon; never have treated fracture of thigh-bone alone, by myself. Have examined plaintiff's thigh before this suit at instance of plaintiff's attorney and defendant, at my office. In supposed cases, and judged actual case, witness differs not essentially from Dr. Trowbridge; he approves of the fracture box; would modify if need there be; six weeks is the usual time for confinement in apparatus, man of 28 years, and good health; as a precaution, might put on lighter dressings after. On inspection at my office, my impression at the time was that the limb was crooked and shortened one-half to three-fourths of an inch; can't say whether from curve or overlapping of bones. (Testimony as to D. White's skill &c. overruled and excepted to by plaintiff.)

Latta's Surgery is a work of authority and standing, not new; Miller's Principles is well spoken of and recent. The American Medical Journal containing Norris' Treatise on Fractures, is good, and posts up to the present day. Statistics of Massachusetts General Hospital, by Norris, also good. Ferguson's practice of Surgery, good; and Samuel Cooper's Surgery is a work of authority. The profession acquire their learning from these books.

I have seen better limbs than Tims', and worse, for strength and usefulness it is about a medium cure. It is a difficult bone to heal, to cure and have it straight.

Know Dr. Parker, of New York; have for past twelve years; has always stood high as a man of authority in surgical matters. Is a Professor and teacher of Surgery in College of Physicians and Surgeons, and has been ever since my acquaintance with him.

Dr. JOSIAH BARNES, sworn: Has examined plaintiff's leg, I came to the conclusion that the curve was sufficient to account for shortening; the cure is about a medium one; has been in the habit of seeing Desault's, Gibson & Hagedorn's apparatus used; saw Cooper's box used only once, and then it was unsuccessful; makes the first, usually, the permanent dressing; calls once in every two or three days. Hypothetical cases endorsed as by Dr. Trowbridge, generally. Has never treated a fracture of thigh bone in and of himself; knows Professor DeLamater, of Cleveland, by reputation; he is an old Professor; he was present at last trial, and stands high as a

Professor. Dr. Wilcox he knows; he has had a good deal of practice and is esteemed a good Surgeon. At the time he was licensed he was so considered.

Dr. NOAH H. WARNER, testifies: He is in practice of medicine—never has had a great practice in Surgery; should use here a straight splint; make a second dressing; calls for first two or three days, two or three times each day, afterwards, once in a day or two; makes second dressing from third to seventh day, is governed by the amount of inflammation; uses 4 splints and bandage 18 tailed,and roller, in place of strings; if oblique fracture would put on long splints. Plaintiff's shortened limb, from examination, I judged to be from lapping of bones. Now, if I were to treat another case I would have the lower splint go under the tuber of the ischium. I differ from other physicians in some things as well as others, I practice homœopathy.

[The plaintiff at this point has called all his witnesses save Dr. Sprague who is confined by sickness. As his testimony was deemed quite essential and was had at two former trials, we refer to minutes of first, and give in brief his peculiarities.]

Dr. A. S. SPRAGUE, sworn: Saw the limb of Tims the day it was fractured, gave it a very slight examination, think it was slightly oblique. If properly dressed and secured, should have been straight now; I think that the bones were not directly in apposition. If limb was in proper position when it came out of the fracture-box, the callus must have been fractured again to put it in its present position. Any bending would break the callus. Almost all the large muscles of the thigh have a tendency to turn the thigh outward, eight-tenths of the power is outward. There is sometimes an evil in keeping the dressing on too long. Muscular action would be likely to break the callus not bend it; if the leg [callus] had been broken by spasmodic action of the muscles, he would not have walked on it a week afterwards. If the bones were properly adjusted there would be no bulge such as is on the plaintiff; I keep on the long splints eight or ten weeks, when I take off splints, I usually keep on some light splints some three or four weeks afterwards. I put them on to guard against spasmodic action, and sometimes to relieve the pain. If the patient, after leg is removed from the box, complained of pain, I should seek the cause and its removal. I have heard the testimony of the witnesses as to the pain of plaintiff. I should not think it prudent to let such a patient go without examination; I should be fearful that something was going wrong.

The strength of the muscles would be very much impaired by being kept fracture-box; it would be impossible to say whether the muscular action might fracture the callus after the leg was taken from the box. It might be broken: healthy bones are sometimes broken so; it would not bend much before breaking.

I use the double inclined plane fracture-box. The principal requisites are that the box may be extended or shortened—the machine here produced possesses these requisites.

If there was a splint bound on a protuberance, it would cause pain and soreness; bones not put in apposition would produce more pain than if in apposition.

[ On comparing this testimony with second trial, it is proper to say, the witness adheres generally to his statements, but expresses a different opinion as to the properties of callus.] [*Here plaintiff rests.*]

J. G. Masten *in Defence*—States the law requires ordinary skill, with ordinary care and dilligence, and if it shall be made to appear that defendant possesses this amount of skill, and exercised that amount of care and dilligence, the jury are bound so to render verdict.

The patient has duties to perform as well as the surgeon, and if he shall have by his carelessness or neglect failed here, the surgeon is exonerated.

It is the duty of the surgeon to reduce fractures, that is to put the bones in apposition, and then resort to some mechanical means to keep the bones in their proper position. This may be done at any time within ten or twelve days; in this time, nature is doing nothing towards uniting the bones, but is making preparation for it. He then gave a lucid account of callus, its properties and mode of formation. In support of these positions, he reads from Latta on Surgery, Miller's Principles of Surgery, Norris, in 3d volume American Medical Journal, new series, also 1st and 4th; Samuel Cooper's Surgery, and Ferguson's Surgery.

Dr. James M. Newman sworn:—Read my profession with Dr. White, and have been in practice one year; attended three courses of lectures and graduated in Cleveland. Recollect Dr. White's attending plaintiff's broken leg, was first informed of the accident by plaintiff's father, who came to Dr. White's office for his aid. Dr. W. was not in; I sought and found him; told him of it, and he rode away. In a little while after Mr. Lane, (Dr. Sprague's student) came in and requested me to go up with the fracture-box to the plaintiff's house. I took it and went up. After the bandages were prepared, Dr. White proceeded to reduce the fracture.

One man held the foot and made gentle extension upon it. Dr. White rolled the limb, with rollers, from the ancle to the thigh. After the extension was made, he put a long splint on the outside of the thigh; this splint is like the one put on (witness points to a splint in court-room) and a shorter one on the inside—these were properly fastened by a roller around them. Dr. White took hold of the thigh at the setting. I was standing at the side of the bed looking on; patient was on the bed; Mr. Lane was in the room. I heard a loud snap at the time the parts were adjusted proceeding from the seat of fracture.

After the splints and rollers were on, that apparatus, (apparatus in court) was put to the well leg and adjusted by its screws, so as to fit the well leg and get it to the right length, it was then placed on the fractured limb and fitted by filling cotton at such places as required to make it comfortable and level, and then strapped up with the buckle. It fitted when it was strapped, nicely to the leg. The patient was left in bed on his back, leg in box and knee bent. The foot board was not in at the time. The fracture was reduced in the morning. I did not examine the limb; had before seen fractures during my pupilage, from July to December; but had never seen a broken thigh or the fracture-box applied before. Dr. White left the patient before dinner, and left town for Geneva in the afternoon. At Dr. White's request, made through me, Dr. Wilcox, a practicing surgeon, and former student of Dr. White, visited the patient in the evening with me, a little after dark, and he put on the foot-board and fastened the foot by bandages. Dr. White was absent a few days and left Dr. Wilcox to take charge of Tims.] [I visited plaintiff next day with Dr. Wilcox; he was comfortable and nothing was done. I saw him again a day or two after; he made no particular complaint; heard of nothing wrong.

I saw him the next time when the apparatus was finally removed by Dr. White, the latter part of the week preceding the 22d of January, cannot remember the exact day. No one was present except Mrs. Tims. It was in the morning betwern 9 and 12. The splints all set snugly upon the thigh at the time, the whole length of it. Dr. White asked how long since the limb was broken. Mrs. Tims made a calculation; it was a little over six weeks. After unfastening the straps, I raised the limb and continued to hold it while the doctor removed the apparatus; the splints appeared to be fastened firmly. I laid the limb upon the bed. Dr. White placed the limbs together the heels and toes; after looking at the limb, the Dr. said it was straight, and told Mr. Tims as he was a mechanic to take sight across the limb and see if it was straight. Tims raised his shoulders and looked at the leg and said it was straight. Mutual congratulations passed between them about the success of the cure. The Dr. told him he must keep in bed and not attempt to get up, and gently use the limb in bed, so as to acquire the use of it again slowly. I looked at the leg and compared it with the other; it appeared to me to be straight. I believe I felt of the callus at the time. I went for the purpose of seeing and learning.

The 22d of January was the next time I knew anything about it. Called at Tims' house with Dr. Wilcox to enquire where Hilliard, who was sick, lived. Dr. Wilcox asked plaintiff how he was getting along. He said he had had some little pain. He did not complain of having had any severe pain. His wife said he had been standing up that day. Dr. Wilcox told him he must not get up, that he must use his limb in bed. This was Monday evening, after dark, the Monday after the leg was taken from the box.

The next time I saw the leg was in Dr. White's office, where Tims came to see about the leg; he walked with a cane, the time I cannot fix upon. I had before heard of the leg's being crooked. Plaintiff and Dr. White went into the side office. Dr. White requested witness and Mr. Dayton, a student, to come in. Dr. W. requested me to get the splint that had been on the outside of plaintiff's leg. Plaintiff was standing on the floor with pantaloons slipped down. Dr. White requested me to place the splint on the thigh in the position it had been formerly; this I did, and plaintiff said it did not set then as before, before it sat flat on the thigh; it did not at this time in consequence of deformity. Conversation then ensued about it. Tims said his thigh was straight when the splint was taken off, spoke of his having severe pain, and of the crook coming on subsequent to his having the pain. He said the pains were in place described, (putting his hand on the inside of the thigh.)

Dr. White went away the 22d of January, and was gone a few days. I do not remember Sarah Maloy's coming to the office on Friday, and asking for Dr. White to come and see Tims; don't know whether Dr. W.'s absence included Friday or not. Mr. Lane is out west in some western state.

Dr. Charles Wilcox, sworn: (Plaintiff objects to his testimony, overruled and exception taken.) Is a Physician and Surgeon; recollects when plaintiff's leg was broken; had seen and treated fractured bones before I saw Tims' leg; some 12 or 15 cases. The first I knew of Tims' case was when Dr. Newman called on me in the evening, to go with him and apply the foot-board; this I did 2d December. Applying the roller I thus fastened the foot to the foot-board; with all the care I could. I saw plaintiff the next day, 2d, 3d, 4th, 5th, and 7th days after. I made it my business to go on these occasions. He appeared to be very comfortable for a man in his situation. On the 7th day I opened the outside splints of the box, made an examination and applied cotton around it, to add to his comfort. Dr. White returned the 7th or 8th December and resumed the charge after that. I do not recollect promising the plaintiff that if defendent did not return on the 7th day I would take off the dressing, aud re-dress it on account of the severe or any pain he had. I heard no unusual pains complained of; don't recollect of the patient's requesti g me to examine the limb. I discovered no inflammation or anything unusual during the time I attended him.

In a few days after Dr. White requested me to assist in re-dressing the limb. I did so. We proceeded to remove the fracture-box and all the dressings on the bear skin. Mrs. Tims, Mr. Deer, and one or two ladies were present, one was Mrs. Hilliard, I believe, I am not positive. Mr. Deer came in for the purpose of assisting. The limb's appearance showed the effect of the bandaging. It was but little, if any, swollen. The leg was washed off with whiskey and water and laid upon the bed. In taking it out of box, Deer held the heel; I supported the thigh. No complaint of pain attracted my a'tention. At time of dressing it would not be unusual to complain of pain. The bones remained in apposition, perfectly, while I was around there; the leg appeared at time perfect and straight; I saw no deformity. Dr. White adjusted the bandages, fracture-box and splints; I held the thigh; all was done under my immediate eye. We then left.

I saw it again the 19th January. It was Friday; know from dates on my book. I understood it to be the day after it was taken from the box. Dr. White was with me. We had a conversation, as also Tims, about it. I recollect examining the leg as plaintiff lay in bed, the fracture-box was removed; Dr. White also examined it. My attention was called to the callus; we were conversing about the strength of the callus to support the limb, I felt of it, raised the limb by the heel, found the leg straight, did not test its strength any other way. The legs were examined as to length. I saw no difference after taking particular notice. This was a surgical examination to discover the length of the limb. Tims seemed to congratulate himself, he said it was a good job. The substance of the language was that he was well pleased with the result. We were there ten or fifteen minutes and had no other business except to see the leg. I did not hear it said, intimated from any source, or suspect it was crooked. I heard no complaints, he appeared to enjoy good health, inquired how long before he could get up. Dr. White told him he could go out in two or three weeks. He directed a liniment to be had at his office to rub his limb, also directed him to flex and extend it and to remain in bed. I considered his limb in a condition then to be dismissed without the bandages, &c. The leg was not swollen; the skin was well under the circumstances; it showed the mark of the bandages.

I next saw the limb the Monday evening following, with Dr. Newman; I called to enquire my way to Hilliard's house. Plaintiff was in bed, I raised the clothes and passed my hand over the leg, and gave it a superficial examination. Did not notice any change save that muscles appeared to be getting more natural and circulation had begun. Heard no complaint of pain—had no intimation there was pain; did not suspect or have intimation from any one that it was crooked. Nothing further took place that night. Tims said he had been sitting by the stove that day; had been sitting up; I recollect I chided him for it.

I next saw the leg a week from that day, on Monday. The first Monday, 22d January, there was neither pantaloons or drawers on the leg. On the 29th January, Monday, I saw it with Dr. White. I had heard it was crooked, met the Dr. and told him of it; he took me into his sleigh and carried me up. The 28th I first heard it was crooked. During the week preceding the 29th I had been visiting Hilliard who was sick. On examining the leg on the 29th, we found it crooked. There was a conversation as to how it came so; plaintiff said on the Thursday before, the 28th, he had suffered great pain in his ham-strings and leg and the curve came on simultaneously. We examined the leg and proposed to straighten it. Tims inquired how much he would suffer from the pain we explained to him, and that was not raised as an objection. He asked how much shorter the limb would be. Dr. White suggested a high boot-heel would relieve it. I urged him to have the attempt made to re-adjust it. He refused. I think the opposition to having it done, was from both him and his wife. We explained all about it; It was the 29th January. The process of extension would not necessarily have fractured the callus. I think we laid no splint upon it. The conclusion was, that if the callus was bent by spasms or other cause it might be restored. I think it would have been injurious to have put a splint on the outside, one might have been put on the inside; on the 19th I saw nothing to prevent the limb from supporting itself, if the patient was kept in bed. I should not have allowed the patient to get up. Druitt's Surgery is good authority.

Dr. Lewis P. Dayton, sworn:—Practices at Black Rock, recollects plaintiff coming to Dr. White's office in March. Dr. Newman was present and Dr. White. Dr. N. got a splint, which they said was the one which had been on plaintiff's leg; the splint was like the one shown in court. Plaintiff said the splint fitted the leg at the time the box was removed, that some two or three days after the box was removed he had violent spasms and cramps in his thigh; indicated where, by putting his hand on the inside of the thigh, and that about that time he discovered a bunch on the outside of his thigh. There was something said about his getting out of bed, or turning his feet off the edge of the bed, or getting up. I recollect that Dr. White said you know that was contrary to directions. Tims assented. The conversation took place chiefly between Newman, Tims and myself Dr. W. having been called into another room to see a patient. I recollect plaintiff said that he sighted across the leg when it came out, and it was straight.

Dr. Robert L. Lane, *de bene esse*, Sept. 1845—read. Deposes:—He is a Physician and Surgeon, former pupil of Drs. Sprague and Loomis—knows the parties to this suit. Was present in the fall of 1843, when Dr. White reduced fracture of thigh for Wm. Tims: had been two years and nearly a half a student of medicine at this time; went to Tims's house with Dr. Sprague; paid particular attention to this operation because Dr. White, was the surgeon. Viewed the limb and its deformity; deformity was removed by the operation, and I knew the bones of the leg were put in apposition by the snapping noise made on their coming together, a noise as if the bones had come suddenly together; have witnessed the setting of legs before, but did not then hear a snapping noise. I was paying particular attention in plaintiff's case; stood close beside the bed. The leg was bare, and after the operation was performed, I observed that the limb had come to its natural shape; the unnatural bend or crook of the limb was removed; saw the limb well dressed and put into the fracture-box; witness here makes a statement of his going for the fracture-box and not obtaining its foot-board. From the circumstance that Dr. Sprague is an eminent surgeon, have had better opportunities than most students to acquire a knowledge of surgery.

Alanson Webster sworn: Knows the plaintiff and his father; at the time the leg was broken they were in my employ; had a conversation with the father after the leg was taken out of the box. He said the leg was as straight, or as good as the other, I can't tell which. He was at the Depot at the time of the conversation. He used to speak about the leg pretty much every morning when he came to work. He told me the day before, that the leg was to be taken out of the box, and the next morning we talked about it. At a subsequent time he told me either it was, or he was afraid it was crooked. Before the injury, plaintiff was a smart active man.

Gilbert Hoyt sworn: testifies he knows the plaintiff and defendant. I saw the limb set; it was deformed before it was set, I saw it go together; heard the bones snap as they went together, and saw the deformity disappear; I stood at the foot of the bed; saw the plaintiff next Sunday, also about a fortnight after second dressing; he appeared to be comfortable, I heard no complaints. I know old Mr. Tims; the next morning after leg came out, he was inquired of at the Depot, how it was; he said the plaintiff had a good leg, and straight—nearly as good as the other; these were the words; the day of the week I cannot tell. Ten days after, I had another talk with old Mr. Tims, he said he was afraid the plaintiff had a bad leg. He said he was a very heavy man, and he was afraid he had used it a little too much.

Harriet W. Doty, *de bene esse*, June 26, 1844, read. Being duly sworn, says, she knows the parties in this suit. Some time last winter plaintiff broke his leg and defendant attended him as his physician; she lived the second door from plaintiff, was in his house to see him several times; once or twice while the leg was in the cradle; he said he was getting along pretty well, but complained of a good deal of pain; was there on a Thursday after the leg had been taken out of the cradle, in which it had been placed; inquired of plaintiff if it was straight; he replied, yes it was. Witness then asked him how he knew or how he could tell, to which plaintiff replied that it was easy for any one to tell it was straight, for it had all fallen away, and there was not much else of it but skin and bone. Plaintiff said the leg had been in the cradle seven weeks, his wife said it was six weeks and some days. The leg was taken out on Thursday morning; this conversation occurred on Thursday afternoon. No one was in the room but plaintiff, his wife and witness. About a week or ten days after, witness saw the plaintiff again, his leg was then crooked; he then said that he had had a great deal of pain in his leg, and had had violent spasms, thought it very singular, did'nt know the cause, and that the spasms had occurred since witness last saw him.

Dr. Harry W. Bissel sworn: Is a practicing physician and surgeon; the first I ever heard of the case, Dr. White and plaintiff came to my office; Dr. White said he wanted me to examine plaintiff's leg if I had leisure, he had treated it sometime before, Tims was satisfied with the treatment, some of his friends were not. He wished me to examine it critically and give my opinion as to the propriety of the course that had been pursued with it. I heard Mr. Tims's story about the treatment while I was examining it. From what I then could learn of the treatment, I supposed there had been a judicious course pursued, and that when the leg was taken from the splint it was straight. Why I came to this conclusion was from what Tims told me. I remember asking him how he knew the leg was straight when it came from the splints; he raised his leg and said, I gunned it. I don't recollect of any thing being said of his getting up. I made up my mind from Tims's own story; there was no discrepancy in the parties statements. I don't recollect what time Tims said the pain came on after it was taken out of box; it was within a few days, perhaps a day or so, but I can't say. He said he was in great distress and sent for the doctor. If I had been sent for under such circumstances, should have endeavored to discover the reason of the pain; I would probably have examined the limb and given some liniment or anodyne.

Dr. Austin Flint, sworn: knows the parties to this suit. Was called upon at his office for purposes, as testifies Dr. Bissel; the general statement of the case by the parties, as testified is substantially the same. Did not examine or see the limb with the clothes off. The parties did not disagree in making their statements. The plaintiff affirmed distinctly the limb was straight when it came out of the box; I asked him two or three times; nothing was said about the patient having been up. Plaintiff stated the curve came on a few days after box was removed; can't state the precise time, one, two or three days. To question previously put. If a man should get up—sit in a chair at this time, with help of a feeble woman, or alone, the risk and danger would be proximate and imminent, and it would be very injudicious; I should think it might be probably bent at that time, the callus not being very solid. If he got up, a knowledge of this would be of essential service to me in my subsequent treatment :If man of age and constitution of plaintiff, I should say in six weeks dressings might be removed without danger, if the patient continued to be in bed and practiced gently flexion of limb; think it important not to keep dressings on too long as they retard the circulation; debility of the muscles and stiffness of joints might ensue. Healthy formation of callus, &c., depends upon the health of the adjacent parts. I know that callus is flexible, in some cases, after the dressing is off, (6 or 7 weeks); I have seen such a case. I should consider plaintiff's leg about a medium cure; the fracture of the thigh bone is one of the most difficult bones to treat, perhaps the most so. I don't think there is shortness enough for an over-lapping.

The inclined plane and straight splint are both used, and by eminent Surgeons. I use Cooper's double inclined plane. French Surgeons sometimes dress without any short splints. One uses no splints at all and he is an eminent Surgeon; they use extension but no splints.

Dr. Horace A. Ackley, *de bene esse,* Oct. 15th, 1844. Has resided in Cleveland for past 2 years; is Prof. of Surgery in Medical College there; for 8 years previous was a Professor in Willoughby University, Ohio; am at present entirely engaged in practice of Surgery; have not been for many years without cases of fracture constantly under treatment.

In answer to the question what is the proper treatment of a simple fracture of the thigh-bone in a healthy subject:—In the first place I would reduce the fracture; if there were displacement, then place upon the limb some easy and convenient apparatus to keep the fracture reduced or the bones in apposition. I would then see the patient occasionally until I was convinced that union had taken place to a sufficient extent to maintain the fractured extremities in apposition. I would then remove the dressings, use friction, various stimulating liniments, and have the patient gradually accustom the limb to motion.

I consider Sir Astley Cooper's fracture box, a very useful apparatus for fractures, oblique or transverse, of the thigh-bone, and with the splints and their proper application, all that is necessary in the treatment.

I should undress the limb, in from five to ten days, depending upon circumstances; sometimes not at all. If everything now is found favorable, I can see no motives for another dressing at all. I should call from time to time, up to 8th day to examine the limb; if the fracture was complicated with contusion of the soft parts, and swelling, or subsequent inflammation was to be apprehended, spasms, or some general febrile excitement, with a great variety of other untoward symptoms; otherwise, I should pay but little attention, if any, for the first eight days. If no untoward symptoms should arise I should not re-dress, but remove the apparatus between the fourth and sixth weeks after the reduction, and if union had not taken place, should re-apply them, and keep them applied until it had taken place. In answer, generally, I should say from fourth to sixth weeks under ordinary circumstances it is usually safe to entirely remove the apparatus and splints. The patient generally begins to use the limb before bony union has taken place, the provisional callus keeping the bones in their place. Pain is usual after the removal of the apparatus. Frictions, and the application of a bandage, if any thing, is all that here is needed. Usually these pains subside, after a few days, by moderate motion and use of the limb. The Surgeon ceases attendance when convinced the apparatus can safely be dispensed with.

In case of curvature, I consider it practicable to straighten it by a judicious applicatiou of apparatus up to the 60th day after fracture. I make this statement from experience; ordinarily I do not conceive there is danger of breaking the provisional callus up to the time mentioned, by endeavoring to straighten a curve of the limb, if the means are judiciously employed. Much however would depend upon the nature and condition of the callus, the amount of the deformity, and the force required to bring the limb to its natural position. Fracture of the callus probably would not occur unless force was used directly for that object.

Dr. F. H. Hamilton, sworn:—Is a teacher in Surgery, commenced teaching in 1835, now teach in the Buffalo Medical University; have examined plaintiff's leg; think it was two or three years since. Drs. Sprague and Delamater were present; it was a very careful examination. I found the thigh shortened about an inch; think there is a little lapping of the bones and some bend. I treat fractures of the thigh differently, depending upon age and circumstances; I have heard the testimony in this case. I should have placed the limb upon double inclined plane, a roller from toes to knee, 18 tailed bandage on the thigh, one splint I should think enough with the side boards and splints of *this* double inclined plane. I should pad the splint to fit; tie these splints with tape, and secure the foot to the foot-board. If there were no untoward symptoms I should not dress it for eight days. Then I would remove all the splints, open the bandages, examine the limb and replace it in the same way again in the box. I should give it supervision till I removed the box, in five or six weeks, if a man age 28; visiting, if nothing untoward, perhaps twice a week; at expiration of this time, I should remove the bandages and splints entirely; and require the patient to keep in bed, for eight or ten days, without putting his toe to the floor. Any other course would be the exception. Ordinarily I should dismiss the patient at this time, seeing him only once or twice in eight or ten days after. If, when I took the limb out of the box, it was straight, and in three or four days it was like Tims', I should attribute it to its being shortened in the box a little, and the crook to its being affected by spasmodic action or using the limb unduly. If told that in a few days after, he had had severe pains, or had got out of bed in two or three days I should ascribe the result to this and the weight used upon it. I think bending might be a result of his getting out of bed alone, or by aid of a feeble woman; pain is not unfrequent after taking off the bandages. On Saturday morning I should not have had an idea that the limb was bent unless I was told by patient, or that he had been up, and should not examine the limb. My impression is that this cure is nearly but not quite an average. Out of eighteen cases which I have measured and therefore know the results, five were shortened two inches; only three were not shortened at all, two were of a child, but I could not say but that they were shortened. These were all oblique fractures. Fractures of the thigh in adults are generally oblique; transverse are uncommon, I have never met with one. In two of the five, the straight splint was used. In one case I unavoidably, left the leg shorter two inches. Surgeons are about equally divided in the use of the long splint or the double inclined plane. Most Surgeons are now of the opinion that oblique fractures of the thigh, in adults cannot generally be united without some shortening.

A crepitus,, is not positive evidence that the bones have been reduced to their place, it is one of the evidences. This crepitus arises from the rubbing the ends of the bones together. It is ordinarily a grating, sometimes a click. I think a snap might be heard as much in an oblique as in a transverse fracture.

I know Druitt's modern surgery; the work is a very good compilation; he wrote it while he was a student, he told me so himself. He is in London. His personal authority here is worth nothing.

I know Chelius' Surgery, and the commentaries by South. I have reveiwed the work; I know South personally; the reputation of the book is good. The text is better authority than South's. I should differ from Chelius if he said that when a leg might be removed from box, the patient might go around on crutches. He is not good authority, on all points—no writer is. (Counsel for plaintiff reads,) I don't consider the authority you read from Chelius is the most prudent. I differ from him in the rule for keeping on bandages. Chelius was a surgeon at Heidle-burgh; a teacher of opthalmic surgery. South is not now a Professor, has been in the Royal College; I should say South exaggerated about the ease of keeping the bones in apposition.

Dr. Willard Parker sworn:—I am a physician and surgeon; have been for 18 or 19 years; I am a teacher of surgery in the University of the State of New York, known as the "College of Physicians and Surgeons." I have had to do with a reasonable number of broken thighs and limbs; I have heard the testimony of Dr. Wilcox, in the case. Judging of the case on the assumption that that testimoy is true, I should say the practice of Dr. White had been good. Treatment depends upon the age and constitution of the patient. I would treat a case of fracture like this:—I should like to arrange the bed and choose my apparatus. I make the first dressing for the purpose of putting the parts in co-aptation and keeping them so. To keep the patient comfortable we apply the bandages and rollers. For the first eight or ten days, I always put patients on the double inclined plane. The splints on the box are generally all the splints that are required, and this is all there is to be done for the first eight or ten days. I should see that the patient was comfortable myself, or have some one see him. I use both the straight and flexed position. This is the flexed position in this box. The second dressing is for the purpose of looking at the limb to see if the bones are properly adjusted, for now is the time; the provisional callus is forming around it to support it. We assist nature until she assists herself. The callus does not begin to form, before the 10th or fifteenth day; it is formed in four or six weeks; we then lay aside the bandages and let nature take care of herself. I would see the patient the next day after the second dressing, and once or twice after; as a general fact, in from four to six weeks, you will find that you can lay aside the apparatus. After that, the patient must be kept still, not stir, but have the limb rubbed, &c. This is a statement of what I should regard as good practice. If the patient is unwell, we know the union is not as soon; any irregularity that exhausts the system, prevents the callus being well formed. In five or six weeks the callus can be flexed without breaking.

I thought the way Dr. Wilcox testified as to the examination made, was a proper way to test the strength of a limb.

If I found all right in six weeks, should not keep on any bandages after the box was taken off; think it good practice to leave the limb naked, as Dr. Wilcox testified at time. The callus in 47 days is as large as my hand, when it is clasped around the bone, it will feel large; it will be about two years, perhaps, that you will see only a feeble and last trace of the callus.

I don't think there was anything wrong in the conduct of Dr. White on Saturday morning, after the limb came out of the box, as detailed by the witnesses. It is just as it should happen, whether I should have looked at the limb or not. I suppose the limb came out straight and was afterwards found crooked; I have heard on Friday, of Hilliard's attempting to turn the patient around; on Saturday night he was sitting up. These things, I should suppose, would cause the spasms, and have a decided effect.

(Witness examines plaintiff's leg.) The well leg is a crooked thigh; don't feel anything like the bones passing by; the bend is in a way that might occur by hanging the leg over a bed—very like.

In children you will generally get an union without shortening; in a well active man you may not; it depends upon the muscles. The oblique fractures in muscular persons, are the most difficult to cure without shortening. Oblique fractures are the most common in middle aged persons; the curved thigh is the most difficult to manage.

I know Druitt's Principles of Surgery. I concur in the opinion expressed by Dr. Hamilton.

(*In cross-examination.*)—If a limb came out of box like plaintiff's, and I knew it had been kept quiet, &c., I should suppose it would required some explanation from the Surgeon. It might be caused without fault of patient or surgeon. If I should find limb like plaintiff's, I should propose to straighten it then, or in a few days; should as likely as not leave it out of the box; should not expect pains in limb in that condition more than if it came out straight. There is often pain; patients complain oftentimes, of decided pain after bandages are taken off.

I should leave a patient who should attempt to get up, as has been described, or I should give him a decided blowing up. I would not allow him to get up or move at all; should think that standing on his leg would be likely to produce severe pain.

*Defence rests.*—Cause is submitted to Jury by counsel, June 30th, and the Judge charged the Jury, as to the points of law, stating (as by counsel for defendant,) that if they were satisfied that the defendent had exercised ordinary skill, and ordinary care, they were to find for him. He then went into a general review of the testimony; stated that in cases like these the medical testimony should have more weight in matters of opinion, than testimony of other witnesses, as they were, it is to be supposed, better informed upon subjects of the nature here presented.

The Jury conferred with each other for a few minutes and returned a verdict for the defendant. W. T.

